# Aquadi-*n*-but­yl(5-methyl­pyrazine-2-carboxyl­ato)tin(IV) methanol solvate

**DOI:** 10.1107/S1600536808016140

**Published:** 2008-06-07

**Authors:** Zhongjun Gao

**Affiliations:** a Deapartment of Chemistry, Jining University, Shandong 273155, People’s Republic of China

## Abstract

In the monomeric title compound, [Sn(C_4_H_9_)_2_(C_6_H_5_N_2_O_2_)_2_(H_2_O)]·CH_3_OH, the Sn atom is seven-coordinate, displaying a distorted penta­gonal bipyramidal SnC_2_N_2_O_3_ geometry with the two C atoms in the axial sites. In the crystal structure, inter­molecular O—H⋯O hydrogen bonds link the complex and solvent mol­ecules into infinite chains.

## Related literature

For general background, see: Gielen *et al.* (1988 ▶). For a related structure, see: Ma *et al.* (2004 ▶).
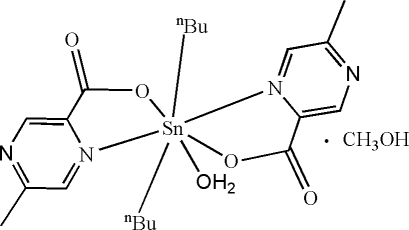


## Experimental

### 

#### Crystal data

[Sn(C_4_H_9_)_2_(C_6_H_5_N_2_O_2_)_2_(H_2_O)]·CH_4_O
*M* *_r_* = 557.21
Monoclinic, 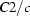

*a* = 20.609 (5) Å
*b* = 17.119 (4) Å
*c* = 14.558 (3) Å
β = 98.178 (3)°
*V* = 5084 (2) Å^3^
*Z* = 8
Mo *K*α radiation
μ = 1.05 mm^−1^
*T* = 298 (2) K
0.58 × 0.56 × 0.49 mm

#### Data collection

Bruker SMART CCD diffractometer
Absorption correction: multi-scan (*SADABS*; Bruker, 1998 ▶) *T*_min_ = 0.582, *T*_max_ = 0.628
12959 measured reflections
4462 independent reflections
2981 reflections with *I* > 2σ(*I*)
*R*_int_ = 0.039

#### Refinement

*R*[*F* ^2^ > 2σ(*F* ^2^)] = 0.036
*wR*(*F* ^2^) = 0.122
*S* = 1.12
4462 reflections
289 parameters
12 restraints
H-atom parameters constrained
Δρ_max_ = 0.58 e Å^−3^
Δρ_min_ = −0.67 e Å^−3^

### 

Data collection: *SMART* (Bruker, 1998 ▶); cell refinement: *SAINT* (Bruker, 1998 ▶); data reduction: *SAINT*; program(s) used to solve structure: *SHELXS97* (Sheldrick, 2008 ▶); program(s) used to refine structure: *SHELXL97* (Sheldrick, 2008 ▶); molecular graphics: *SHELXTL* (Sheldrick, 2008 ▶); software used to prepare material for publication: *SHELXL97*.

## Supplementary Material

Crystal structure: contains datablocks I, global. DOI: 10.1107/S1600536808016140/hb2726sup1.cif

Structure factors: contains datablocks I. DOI: 10.1107/S1600536808016140/hb2726Isup2.hkl

Additional supplementary materials:  crystallographic information; 3D view; checkCIF report

## Figures and Tables

**Table 1:** Selected bond lengths (Å)

Sn1—C17	2.103 (5)
Sn1—C13	2.107 (6)
Sn1—O1	2.161 (4)
Sn1—O3	2.167 (4)
Sn1—N1	2.481 (4)
Sn1—N3	2.635 (5)
Sn1—O5	2.770 (4)

**Table 2:** Hydrogen-bond geometry (Å, °)

*D*—H⋯*A*	*D*—H	H⋯*A*	*D*⋯*A*	*D*—H⋯*A*
O5—H1⋯O6	0.85	1.92	2.755 (6)	169
O5—H2⋯O1^i^	0.85	2.19	3.039 (5)	172
O6—H6⋯O4^i^	0.82	1.93	2.703 (6)	156

Symmetry code: (i) 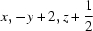.

**Fig. 1.**
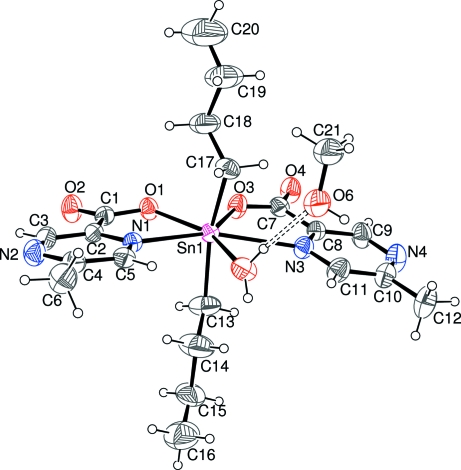
The molecular structure of (I), with displacement ellipsoids for the non-hydrogen atoms drawn at the 30% probability level. The hydrogen bond is indicated by a double-dashed line.

**Fig. 2.**
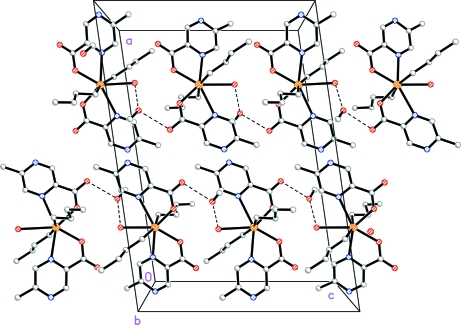
Packing diagram of the crystal structure of (I), showing the one-dimensional chains that form along the c axis. H atoms are omitted for clarity and dashed lines link the donor and acceptor O atoms of the hydrogen bonds.

## supplementary crystallographic information

### Comment

Self-assembled organotin derivatives of carboxylic acid ligands have been extensively studied due to their biological activities (Gielen *et al.*, 1988). 2-Methylpyrazine-5-carboxylic acid is a good bridging ligand that can sometimes be used to generate unexpected and interesting coordination polymers, and small changes in experimental conditions can lead to very different architectures.

The title compound, (I), consists of two butyl, two N,O-bidentate 2-methylpyrazine-5-carboxylate groups and a water molecule bonded to the Sn atom and has a monomeric structure. The Sn atom is seven-coordinate with a distorted pentagonal bipyramidal SnC_2_N_2_O_3_ geometry (Table 1, Fig. 1). Around the central Sn atom, atoms C13 and C17 of the two butyl groups occupy the axial position, while O and N atoms lie in equatorial positions. The sum of the angles between the tin atom and the equatorial atoms is 360.4°. The axial bond angle C17—Sn1—C13 [161.6 (3)°] deviates from linearity by over 18°, which shows that these atoms have significant deviations from ideal pentagonal bipyramidal geometry. Otherwise, the bond lenghts and angles in (I) are similar to those in related structures (Ma *et al.*, 2004).

In the crystal, strong intermolecular O—H···O hydrogen bonds (Table 2) between O atoms of the carboxyl groups, methanol and coordinate water molecules result in the formation of one-dimensional polymeric chains (Fig. 2).

### Experimental

A mixture of dibutyltin dichloride (1.0 mmol, 0.304 g), 2-methylpyrazine-5-carboxylic acid (2.0 mmol, 0.276 g) and sodium ethoxide (0.136 g, 2.0 mmol) in ethanol (with 5% water) (80 ml) was heated under reflux for 8 h at 303 K. The resulting clear solution was evaporated under vacuum and the product recrystallized from a mixture of methanol to yield colourless, blocks of (I). Yield 0.452 g, 78%, m.p. 438 K, analysis, calculated for C_21_H_34_N_4_O_6_Sn: C 45.26, H, 6.15; N 10.05%; found: C 45.39, H 6.29, N, 10.12%.

### Refinement

The H atoms were positioned geometrically (C—H = 0.93–0.97 Å, O—H = 0.82–0.85 Å) and refined as riding with *U*_iso_(H) = 1.2*U*_eq_(C/O) or 1.5U_eq_(methyl C)

### Crystal data

**Table:**

[Sn(C_4_H_9_)_2_(C_6_H_5_N_2_O_2_)_2_(H_2_O)]·CH_4_O	*F*_000_ = 2288
*M**_r_* = 557.21	*D*_x_ = 1.456 Mg m^−^^3^
Monoclinic, *C*2/*c*	Mo *K*α radiation λ = 0.71073 Å
Hall symbol: -C 2yc	Cell parameters from 4606 reflections
*a* = 20.609 (5) Å	θ = 2.4–25.3º
*b* = 17.119 (4) Å	µ = 1.05 mm^−^^1^
*c* = 14.558 (3) Å	*T* = 298 (2) K
β = 98.178 (3)º	Block, colourless
*V* = 5084 (2) Å^3^	0.58 × 0.56 × 0.49 mm
*Z* = 8	

### Data collection

**Table:**

Bruker SMART CCD diffractometer	4462 independent reflections
Radiation source: fine-focus sealed tube	2981 reflections with *I* > 2σ(*I*)
Monochromator: graphite	*R*_int_ = 0.039
*T* = 298(2) K	θ_max_ = 25.0º
ω scans	θ_min_ = 1.6º
Absorption correction: multi-scan(SADABS; Bruker, 1998)	*h* = −15→24
*T*_min_ = 0.582, *T*_max_ = 0.628	*k* = −20→20
12959 measured reflections	*l* = −17→17

### Refinement

**Table:**

Refinement on *F*^2^	Secondary atom site location: difference Fourier map
Least-squares matrix: full	Hydrogen site location: inferred from neighbouring sites
*R*[*F*^2^ > 2σ(*F*^2^)] = 0.036	H-atom parameters constrained
*wR*(*F*^2^) = 0.122	*w* = 1/[σ^2^(*F*_o_^2^) + (0.0455*P*)^2^ + 11.2132*P*] where *P* = (*F*_o_^2^ + 2*F*_c_^2^)/3
*S* = 1.12	(Δ/σ)_max_ = 0.002
4462 reflections	Δρ_max_ = 0.58 e Å^−^^3^
289 parameters	Δρ_min_ = −0.67 e Å^−^^3^
12 restraints	Extinction correction: none
Primary atom site location: structure-invariant direct methods	

### Special details

**Table:**

Geometry. All e.s.d.'s (except the e.s.d. in the dihedral angle between two l.s. planes) are estimated using the full covariance matrix. The cell e.s.d.'s are taken into account individually in the estimation of e.s.d.'s in distances, angles and torsion angles; correlations between e.s.d.'s in cell parameters are only used when they are defined by crystal symmetry. An approximate (isotropic) treatment of cell e.s.d.'s is used for estimating e.s.d.'s involving l.s. planes.
Refinement. Refinement of *F*^2^ against ALL reflections. The weighted *R*-factor *wR* and goodness of fit *S* are based on *F*^2^, conventional *R*-factors *R* are based on *F*, with *F* set to zero for negative *F*^2^. The threshold expression of *F*^2^ > σ(*F*^2^) is used only for calculating *R*-factors(gt) *etc*. and is not relevant to the choice of reflections for refinement. *R*-factors based on *F*^2^ are statistically about twice as large as those based on *F*, and *R*- factors based on ALL data will be even larger.

### Fractional atomic coordinates and isotropic or equivalent isotropic displacement parameters (Å^2^)

**Table:**

	*x*	*y*	*z*	*U*_iso_*/*U*_eq_	
Sn1	0.256428 (17)	1.005653 (19)	0.41391 (2)	0.05022 (15)	
N1	0.3679 (2)	0.9489 (2)	0.4509 (3)	0.0496 (10)	
N2	0.4975 (2)	0.9001 (3)	0.4682 (4)	0.0746 (15)	
N3	0.1453 (2)	1.0475 (3)	0.4696 (3)	0.0597 (12)	
N4	0.0240 (3)	1.1105 (4)	0.4929 (4)	0.0858 (17)	
O1	0.30863 (18)	1.0137 (2)	0.2956 (2)	0.0579 (9)	
O2	0.4017 (2)	1.0160 (3)	0.2351 (3)	0.0784 (12)	
O3	0.18771 (19)	1.0558 (2)	0.3031 (3)	0.0673 (11)	
O4	0.09289 (19)	1.1052 (3)	0.2392 (3)	0.0757 (12)	
O5	0.26009 (19)	0.9499 (2)	0.5931 (3)	0.0736 (11)	
H1	0.2327	0.9135	0.5983	0.088*	
H2	0.2730	0.9647	0.6484	0.088*	
C1	0.3704 (3)	1.0012 (3)	0.2976 (4)	0.0529 (13)	
C2	0.4039 (3)	0.9618 (3)	0.3833 (4)	0.0513 (13)	
C3	0.4686 (3)	0.9380 (4)	0.3940 (4)	0.0679 (16)	
H3	0.4932	0.9491	0.3467	0.082*	
C4	0.4612 (3)	0.8866 (3)	0.5350 (4)	0.0620 (15)	
C5	0.3965 (3)	0.9124 (3)	0.5273 (4)	0.0591 (14)	
H5	0.3727	0.9042	0.5762	0.071*	
C6	0.4918 (3)	0.8426 (4)	0.6191 (4)	0.090 (2)	
H6A	0.5364	0.8300	0.6129	0.135*	
H6B	0.4677	0.7953	0.6250	0.135*	
H6C	0.4910	0.8743	0.6733	0.135*	
C7	0.1295 (3)	1.0804 (3)	0.3063 (4)	0.0586 (14)	
C8	0.1057 (3)	1.0790 (3)	0.3989 (4)	0.0532 (13)	
C9	0.0454 (3)	1.1085 (4)	0.4106 (5)	0.0747 (18)	
H9	0.0183	1.1279	0.3591	0.090*	
C10	0.0627 (3)	1.0788 (4)	0.5628 (5)	0.0722 (17)	
C11	0.1233 (3)	1.0474 (4)	0.5517 (4)	0.0729 (17)	
H11	0.1492	1.0256	0.6027	0.087*	
C12	0.0393 (4)	1.0786 (5)	0.6554 (5)	0.103 (3)	
H12A	−0.0033	1.1022	0.6499	0.155*	
H12B	0.0694	1.1076	0.6989	0.155*	
H12C	0.0368	1.0258	0.6767	0.155*	
C13	0.2915 (4)	1.1117 (4)	0.4756 (5)	0.091 (2)	
H13A	0.3165	1.1364	0.4319	0.110*	
H13B	0.2532	1.1442	0.4778	0.110*	
C14	0.3295 (5)	1.1185 (4)	0.5624 (6)	0.128 (3)	
H14A	0.3671	1.0843	0.5633	0.153*	
H14B	0.3038	1.0990	0.6084	0.153*	
C15	0.3547 (4)	1.1996 (4)	0.5931 (7)	0.111 (3)	
H15A	0.3814	1.2197	0.5488	0.133*	
H15B	0.3178	1.2346	0.5938	0.133*	
C16	0.3937 (5)	1.1977 (5)	0.6860 (7)	0.139 (4)	
H16A	0.4091	1.2494	0.7029	0.208*	
H16B	0.4305	1.1634	0.6854	0.208*	
H16C	0.3670	1.1792	0.7303	0.208*	
C17	0.2167 (3)	0.8926 (3)	0.3962 (4)	0.0697 (17)	
H17A	0.2404	0.8590	0.4431	0.084*	
H17B	0.1716	0.8946	0.4079	0.084*	
C18	0.2177 (4)	0.8561 (4)	0.3060 (5)	0.107 (3)	
H18A	0.2627	0.8497	0.2950	0.129*	
H18B	0.1955	0.8895	0.2577	0.129*	
C19	0.1832 (6)	0.7749 (5)	0.3022 (7)	0.148 (4)	
H19A	0.2050	0.7431	0.3525	0.178*	
H19B	0.1384	0.7826	0.3133	0.178*	
C20	0.1821 (7)	0.7340 (7)	0.2212 (9)	0.217 (7)	
H20A	0.1597	0.6853	0.2258	0.326*	
H20B	0.2262	0.7240	0.2103	0.326*	
H20C	0.1596	0.7640	0.1707	0.326*	
C21	0.1267 (4)	0.7823 (4)	0.5608 (5)	0.104 (3)	
H21A	0.1147	0.7483	0.6081	0.156*	
H21B	0.1551	0.7550	0.5249	0.156*	
H21C	0.0879	0.7986	0.5210	0.156*	
O6	0.1586 (3)	0.8466 (3)	0.6014 (4)	0.123 (2)	
H6	0.1340	0.8703	0.6312	0.147*	

### Atomic displacement parameters (Å^2^)

**Table:**

	*U*^11^	*U*^22^	*U*^33^	*U*^12^	*U*^13^	*U*^23^
Sn1	0.0454 (2)	0.0530 (2)	0.0517 (2)	0.00077 (18)	0.00511 (16)	−0.00375 (17)
N1	0.048 (2)	0.050 (2)	0.051 (3)	0.001 (2)	0.006 (2)	−0.002 (2)
N2	0.057 (3)	0.092 (4)	0.074 (3)	0.017 (3)	0.004 (3)	0.000 (3)
N3	0.055 (3)	0.062 (3)	0.062 (3)	0.010 (2)	0.010 (2)	0.000 (2)
N4	0.066 (3)	0.111 (5)	0.084 (4)	0.014 (3)	0.024 (3)	0.010 (3)
O1	0.046 (2)	0.077 (2)	0.050 (2)	0.0019 (19)	0.0050 (17)	0.0063 (18)
O2	0.065 (3)	0.114 (3)	0.059 (3)	0.007 (2)	0.018 (2)	0.018 (2)
O3	0.056 (2)	0.088 (3)	0.058 (2)	0.019 (2)	0.0097 (19)	0.005 (2)
O4	0.057 (2)	0.110 (3)	0.058 (3)	0.018 (2)	0.002 (2)	0.011 (2)
O5	0.072 (3)	0.078 (3)	0.069 (3)	−0.014 (2)	0.003 (2)	0.000 (2)
C1	0.053 (3)	0.058 (3)	0.047 (3)	0.001 (3)	0.007 (3)	−0.003 (3)
C2	0.049 (3)	0.056 (3)	0.049 (3)	0.004 (3)	0.006 (3)	−0.007 (2)
C3	0.061 (4)	0.083 (4)	0.061 (4)	0.012 (3)	0.012 (3)	−0.006 (3)
C4	0.061 (4)	0.058 (3)	0.063 (4)	0.009 (3)	−0.005 (3)	−0.001 (3)
C5	0.058 (4)	0.064 (3)	0.054 (3)	−0.002 (3)	0.002 (3)	−0.007 (3)
C6	0.087 (5)	0.103 (5)	0.077 (5)	0.022 (4)	0.000 (4)	0.009 (4)
C7	0.051 (3)	0.063 (3)	0.060 (4)	0.000 (3)	0.001 (3)	−0.004 (3)
C8	0.048 (3)	0.054 (3)	0.058 (3)	0.002 (3)	0.007 (3)	−0.004 (3)
C9	0.052 (4)	0.096 (5)	0.075 (4)	0.011 (3)	0.006 (3)	0.009 (4)
C10	0.072 (4)	0.076 (4)	0.073 (4)	0.006 (4)	0.024 (4)	0.002 (3)
C11	0.074 (4)	0.082 (4)	0.062 (4)	0.016 (4)	0.010 (3)	0.001 (3)
C12	0.101 (6)	0.129 (7)	0.090 (5)	0.021 (5)	0.048 (5)	0.011 (5)
C13	0.122 (6)	0.059 (4)	0.084 (5)	0.006 (4)	−0.020 (5)	−0.013 (3)
C14	0.136 (8)	0.087 (6)	0.148 (8)	−0.019 (5)	−0.023 (7)	−0.027 (5)
C15	0.102 (6)	0.070 (5)	0.157 (8)	−0.022 (4)	0.007 (6)	−0.034 (5)
C16	0.165 (10)	0.097 (6)	0.153 (9)	−0.007 (6)	0.016 (8)	−0.028 (6)
C17	0.071 (4)	0.053 (3)	0.083 (4)	−0.013 (3)	0.005 (3)	−0.003 (3)
C18	0.131 (7)	0.083 (5)	0.108 (6)	−0.028 (5)	0.021 (5)	−0.037 (4)
C19	0.191 (11)	0.111 (7)	0.142 (9)	−0.040 (7)	0.023 (8)	−0.055 (6)
C20	0.265 (17)	0.138 (10)	0.244 (16)	−0.054 (11)	0.022 (13)	−0.074 (11)
C21	0.115 (6)	0.093 (5)	0.107 (6)	−0.020 (5)	0.028 (5)	−0.019 (5)
O6	0.118 (4)	0.129 (4)	0.134 (4)	−0.045 (4)	0.065 (3)	−0.047 (3)

### Geometric parameters (Å, °)

**Table:**

Sn1—C17	2.103 (5)	C10—C12	1.494 (9)
Sn1—C13	2.107 (6)	C11—H11	0.9300
Sn1—O1	2.161 (4)	C12—H12A	0.9600
Sn1—O3	2.167 (4)	C12—H12B	0.9600
Sn1—N1	2.481 (4)	C12—H12C	0.9600
Sn1—N3	2.635 (5)	C13—C14	1.392 (9)
Sn1—O5	2.770 (4)	C13—H13A	0.9700
N1—C2	1.331 (6)	C13—H13B	0.9700
N1—C5	1.337 (6)	C14—C15	1.526 (8)
N2—C3	1.326 (7)	C14—H14A	0.9700
N2—C4	1.330 (8)	C14—H14B	0.9700
N3—C8	1.333 (6)	C15—C16	1.473 (10)
N3—C11	1.337 (7)	C15—H15A	0.9700
N4—C10	1.317 (8)	C15—H15B	0.9700
N4—C9	1.334 (8)	C16—H16A	0.9600
O1—C1	1.288 (6)	C16—H16B	0.9600
O2—C1	1.215 (7)	C16—H16C	0.9600
O3—C7	1.278 (6)	C17—C18	1.456 (8)
O4—C7	1.223 (6)	C17—H17A	0.9700
O5—H2	0.8500	C17—H17B	0.9700
O5—H1	0.8500	C18—C19	1.558 (9)
C1—C2	1.498 (7)	C18—H18A	0.9700
C2—C3	1.383 (7)	C18—H18B	0.9700
C3—H3	0.9300	C19—C20	1.369 (11)
C4—C5	1.395 (8)	C19—H19A	0.9700
C4—C6	1.498 (8)	C19—H19B	0.9700
C5—H5	0.9300	C20—H20A	0.9600
C6—H6A	0.9600	C20—H20B	0.9600
C6—H6B	0.9600	C20—H20C	0.9600
C6—H6C	0.9600	C21—O6	1.372 (8)
C7—C8	1.498 (8)	C21—H21A	0.9600
C8—C9	1.376 (8)	C21—H21B	0.9600
C9—H9	0.9300	C21—H21C	0.9600
C10—C11	1.390 (8)	O6—H6	0.8200
			
C17—Sn1—C13	161.7 (3)	N4—C10—C12	117.4 (6)
C17—Sn1—O1	100.9 (2)	C11—C10—C12	121.0 (6)
C13—Sn1—O1	96.1 (2)	N3—C11—C10	121.9 (6)
C17—Sn1—O3	94.1 (2)	N3—C11—H11	119.0
C13—Sn1—O3	97.1 (2)	C10—C11—H11	119.0
O1—Sn1—O3	74.24 (14)	C10—C12—H12A	109.5
C17—Sn1—N1	89.9 (2)	C10—C12—H12B	109.5
C13—Sn1—N1	89.6 (2)	H12A—C12—H12B	109.5
O1—Sn1—N1	69.34 (14)	C10—C12—H12C	109.5
O3—Sn1—N1	143.46 (14)	H12A—C12—H12C	109.5
C17—Sn1—N3	87.0 (2)	H12B—C12—H12C	109.5
C13—Sn1—N3	84.0 (2)	C14—C13—Sn1	125.0 (5)
O1—Sn1—N3	141.33 (14)	C14—C13—H13A	106.1
O3—Sn1—N3	67.44 (15)	Sn1—C13—H13A	106.1
N1—Sn1—N3	149.10 (14)	C14—C13—H13B	106.1
C17—Sn1—O5	75.89 (18)	Sn1—C13—H13B	106.1
C13—Sn1—O5	86.2 (2)	H13A—C13—H13B	106.3
O1—Sn1—O5	145.38 (13)	C13—C14—C15	117.7 (7)
O3—Sn1—O5	139.93 (13)	C13—C14—H14A	107.9
N1—Sn1—O5	76.15 (13)	C15—C14—H14A	107.9
N3—Sn1—O5	73.29 (13)	C13—C14—H14B	107.9
C2—N1—C5	117.8 (5)	C15—C14—H14B	107.9
C2—N1—Sn1	111.8 (3)	H14A—C14—H14B	107.2
C5—N1—Sn1	130.4 (4)	C16—C15—C14	111.6 (7)
C3—N2—C4	116.5 (5)	C16—C15—H15A	109.3
C8—N3—C11	116.3 (5)	C14—C15—H15A	109.3
C8—N3—Sn1	109.6 (3)	C16—C15—H15B	109.3
C11—N3—Sn1	134.0 (4)	C14—C15—H15B	109.3
C10—N4—C9	116.3 (6)	H15A—C15—H15B	108.0
C1—O1—Sn1	125.1 (3)	C15—C16—H16A	109.5
C7—O3—Sn1	128.4 (4)	C15—C16—H16B	109.5
H2—O5—Sn1	138.8	H16A—C16—H16B	109.5
Sn1—O5—H1	113.9	C15—C16—H16C	109.5
Sn1—O5—H2	138.8	H16A—C16—H16C	109.5
H1—O5—H2	105.0	H16B—C16—H16C	109.5
O2—C1—O1	125.3 (5)	C18—C17—Sn1	116.8 (4)
O2—C1—C2	119.2 (5)	C18—C17—H17A	108.1
O1—C1—C2	115.5 (5)	Sn1—C17—H17A	108.1
N1—C2—C3	119.9 (5)	C18—C17—H17B	108.1
N1—C2—C1	116.8 (5)	Sn1—C17—H17B	108.1
C3—C2—C1	123.3 (5)	H17A—C17—H17B	107.3
N2—C3—C2	123.4 (6)	C17—C18—C19	110.5 (6)
N2—C3—H3	118.3	C17—C18—H18A	109.6
C2—C3—H3	118.3	C19—C18—H18A	109.6
N2—C4—C5	121.1 (5)	C17—C18—H18B	109.6
N2—C4—C6	118.0 (5)	C19—C18—H18B	109.6
C5—C4—C6	120.9 (6)	H18A—C18—H18B	108.1
N1—C5—C4	121.2 (6)	C20—C19—C18	116.0 (9)
N1—C5—H5	119.4	C20—C19—H19A	108.3
C4—C5—H5	119.4	C18—C19—H19A	108.3
C4—C6—H6A	109.5	C20—C19—H19B	108.3
C4—C6—H6B	109.5	C18—C19—H19B	108.3
H6A—C6—H6B	109.5	H19A—C19—H19B	107.4
C4—C6—H6C	109.5	C19—C20—H20A	109.5
H6A—C6—H6C	109.5	C19—C20—H20B	109.5
H6B—C6—H6C	109.5	H20A—C20—H20B	109.5
O4—C7—O3	124.2 (6)	C19—C20—H20C	109.5
O4—C7—C8	118.7 (5)	H20A—C20—H20C	109.5
O3—C7—C8	117.2 (5)	H20B—C20—H20C	109.5
N3—C8—C9	121.1 (5)	O6—C21—H21A	109.5
N3—C8—C7	117.3 (5)	O6—C21—H21B	109.5
C9—C8—C7	121.6 (5)	H21A—C21—H21B	109.5
N4—C9—C8	122.8 (6)	O6—C21—H21C	109.5
N4—C9—H9	118.6	H21A—C21—H21C	109.5
C8—C9—H9	118.6	H21B—C21—H21C	109.5
N4—C10—C11	121.6 (6)	C21—O6—H6	109.3

### Hydrogen-bond geometry (Å, °)

**Table:**

*D*—H···*A*	*D*—H	H···*A*	*D*···*A*	*D*—H···*A*
O5—H1···O6	0.85	1.92	2.755 (6)	169
O5—H2···O1^i^	0.85	2.19	3.039 (5)	172
O6—H6···O4^i^	0.82	1.93	2.703 (6)	156

Symmetry codes: (i) *x*, −*y*+2, *z*+1/2.
